# Assessment of Local and Regional Control in High-Risk Atypical (WHO Grade 2) Meningiomas Receiving Stereotactic Fractionated Radiosurgery

**DOI:** 10.7759/cureus.77322

**Published:** 2025-01-12

**Authors:** Roman Travis, Madyson Taylor, Christopher Willey, Markus Bredel, Kristen O Riley, James M Markert, John Fiveash

**Affiliations:** 1 Radiation Oncology, University of Alabama at Birmingham School of Medicine, Birmingham, USA; 2 Neurological Surgery, University of Alabama at Birmingham School of Medicine, Birmingham, USA

**Keywords:** brain, local control, meningioma, radiosurgery, resection cavity

## Abstract

Purpose

Adjuvant radiation therapy for atypical meningiomas (AMs) aids in local control following surgery and salvage after recurrence. The role of fractionated stereotactic radiosurgery (FSRT) in this population remains an area of active study with many unanswered clinical questions. This single-institution retrospective study evaluates the local control, marginal control, and toxicity of FSRT in treating AM.

Methods

Between 2009 and 2022, 39 patients with WHO grade 2 AM underwent FSRT via marginless, frameless volumetric-modulated arc therapy (VMAT) at doses of 27.5-30 Gy in five fractions. Local recurrence was defined as an increase of 20% in the greatest cross-sectional diameter on MRI or CT, following RECIST criteria. Cavity and marginal recurrences were defined as any new lesion outside the prescription volume but within the resection cavity or within 2 cm of the resection cavity, respectively. High-grade toxicity was defined per Common Terminology Criteria for Adverse Events (CTCAE) v5. Resection for radionecrosis with viable residual tumor was considered a local failure.

Results

Twenty-six AMs were treated post-subtotal resection (STR), 16 post-gross total resection (GTR) with recurrence, and five treated definitively. Patient characteristics included a mean age of 54 years, 20 (51%) male patients, and 31 (79%) patients with ECOG 0-1. The three-year local control rate was 84.0%. Larger tumors were more likely to fail locally (p > 0.001). Two (5%) patients experienced high-grade toxicity necessitating resection. The three-year marginal control rate was 92.3%, and recurrent tumors post-GTR failed marginally more often compared to those treated after STR (p = 0.009). One (4%) tumor treated after STR failed marginally, while four (33%) tumors treated after GTR recurrence failed marginally. The three-year control rate of the unirradiated cavity was 88%.

Conclusion

The rate of high-grade toxicity in AM patients receiving FSRT was low. Local control appeared comparable to historical rates, which may suggest the potential need for dose escalation with longer-term follow-up. Recurrent tumors were more prone to marginal failures. Further investigation is needed to determine which patients may benefit from whole-cavity treatment, additional CTV margin, or prolonged fractionated dose schedules. Newer imaging studies, including DOTATATE PET, should be explored to assess whether improvements in targeting accuracy can enhance outcomes.

## Introduction

The role of adjuvant radiation in the treatment of atypical meningiomas (AMs) remains a topic of ongoing debate [1-3]. While maximal safe resection is the initial standard of care, the dose and timing of adjuvant/salvage radiation are determined by clinical factors, including the extent of resection, life expectancy, and histological characteristics.

In cases of subtotal resection (STR), high-dose adjuvant radiation is necessary to achieve even moderate tumor control [4]. For instance, in RTOG 0539, combined analysis of recurrent and subtotally resected AMs showed a three-year progression-free survival of 35.7% with radiation doses ranging from 54 to 60 Gy [5]. In a retrospective study of recurrent AMs, prior STR was associated with worse overall survival (HR = 1.4-10.5) [6]. Given the high recurrence rates and worse survival, the NCCN recommends adjuvant radiation for AMs after STR [7].

There is less consensus on the role of adjuvant radiation in AMs after GTR. A previous retrospective study at this institution reported a local recurrence rate of 30.3% at three years in AMs after GTR alone [8]. In contrast, the single-arm EORTC 22042-26042 study found an impressive three-year progression-free survival of 88.7% in AMs treated with 60 Gy after GTR [9]. In a 2018 retrospective study of recurrent AM, each round of salvage therapy was associated with greater morbidity with reduced control, possibly due to increasingly aggressive growth characteristics as observed in histology [6]. While these findings suggest a potential benefit of upfront adjuvant radiation, there is also support for delaying radiation in favor of using it in the salvage setting. The BN003 study is currently evaluating the omission of radiation in AMs after GTR [10].

Stereotactic radiosurgery (SRS) has long been recognized as highly effective and safe for treating grade 1 meningiomas [11,12]. Its use in higher-grade meningiomas has historically been limited, often reserved for salvage treatment, due to high rates of recurrence and toxicity [13,14]. Traditionally, higher-grade tumors are treated with fractionated radiation therapy, utilizing large clinical target volume (CTV) margins to encompass potential invasion into normal brain parenchyma. While concerns for increased normal brain invasion in higher-grade meningiomas are valid, it is important to note the recent migration of WHO staging, with many tumors being upgraded from WHO grade 1 to 2 in 2007 and 2021 [15]. Given this staging shift and the increased use of fractionated stereotactic radiation therapy (FSRT) with linear accelerator (LINAC)-based systems, FSRT deserves more attention as a treatment option for AMs. Retrospective studies have demonstrated that FSRT provides good local control in AM, though careful dose limitations are necessary in high-risk areas, such as the optic nerves [16]. One retrospective study found a grade 3+ toxicity rate as high as 27.1% in AMs treated with single-fraction SRS [17]. Currently studied in brain metastases, FSRT offers excellent tumor control with less concern for toxicity. Unfortunately, data on this technique in AM are limited to small retrospective cohorts, often included with fractionated patients [4]. Stereotactic techniques typically involve smaller CTV and planning target volume (PTV) margins, raising the question of whether FSRT may result in a higher rate of marginal recurrences compared to the larger volumes used in conventionally fractionated therapy.

This single-institution retrospective study examines the patterns of recurrence and toxicity in AMs treated with FSRT, with the aim of aiding physicians in making treatment decisions by providing underreported data on local control and toxicity outcomes. Failure patterns to be examined include local control, recurrence in untreated cavities, and recurrence within a 2-cm margin, the largest used in RTOG 0539.

The hypothesis of this study is that FSRT will provide excellent local control of AMs, achieve low-cavity failure rates compared to historical controls, and result in acceptable toxicity.

This article was previously presented as a poster at the 2024 ASTRO American Society for Radiation Oncology Annual Meeting on September 29, 2024.

## Materials and methods

This retrospective review included all patients with cranial AMs treated with FSRT between 2009 and 2022 at the University of Alabama at Birmingham. Tumor grading was based on the WHO criteria active at the time of treatment. Tumors eligible for inclusion were initially determined according to the RTOG 0539 definition of high-risk meningiomas: any grade 3 post-GTR or STR, recurrent grade 2 post-GTR or STR, or new grade 2 post-STR. This classification schema was subsequently modified to exclude any tumor with grade 3 histology, leaving only those with grade 2 classifications. In addition, any tumor that had previously received fractionated EBRT or any patient treated adjuvantly after GTR was excluded from the study. All patients had radiographically confirmed gross tumor present at the time of treatment.

Radiosurgery

All radiation treatments were delivered using frameless volumetric-modulated arc therapy (VMAT) with zero PTV margin. Inclusion criteria required a histological diagnosis of AM, the presence of gross disease at the time of FSRT, a five-fraction FSRT, and no prior local radiation. Single-fraction treatments were excluded from this analysis. FSRT was chosen due to the larger treatment volume and the proximity to very high-risk structures, as determined by physician preference. Multi-fraction treatment was preferred for recurrences greater than 2-3 cm or located in unfavorable regions. According to institutional practice, CTV included all visible gross tumors, without a significant elective dural tail volume. CTV was not expanded with a margin into normal brain tissue, as is common in standard fractionated approaches. Definitions for target volumes are provided in Table 1 for reference. Only five patients in this study received whole-cavity treatment, defined as the radiation of the entire resection cavity along with the recurrent gross tumor volume (GTV). CTV was treated without a PTV margin. Notably, the practice at this institution during the study period was to generally omit radiation for most AM patients who underwent GTR.

**Table 1:** Comparison of progression-free survival and dosing in previous studies investigating meningioma radiation therapy. STR: subtotal resection, CTV: clinical target volume, GTV: gross tumor volume, Gy: gray, fx: fractions, PFA: progression-free survival.

	NRG-BN003 [10]	RTOG 0539 GTR [5]	RTOG 0539 recurrent/subtotal resection [5]	This study
Patients (n)	148 (goal)	36	29	39
Endpoint	PFS	Three-year PFS	Three-year PFS	Three-year PFS locally and marginally
Target definition	Tumor bed + 0-5 mm CTV	Tumor bed + 5-10 mm CTV	CTV60, GTV + 10 mm; CTV54, GTV + 20 mm	Gross disease + tumor bed per physician preference
Dose schedule	59.4 Gy/33 fx	54 Gy in 30 fx	54/60 Gy in 30 fx	27.5-30 Gy/5 fx

Patients were immobilized using a thermoplastic mask. Planning was performed by registering a contrast-enhanced CT simulation to a contrast-enhanced MRI. The prescribed dose was delivered without a PTV margin, such that 99% of the GTV received 100% of the prescription dose (V100% 99%). Tumors were treated with 27.5-30 Gy over 7-14 calendar days. The dose was delivered in 2-4 single-isocenter VMAT arcs, using either the Varian Edge Radiosurgery (Varian Medical Systems, Inc., Palo Alto, USA) platform or the TrueBeam STx (Varian Medical Systems, Inc., Palo Alto, USA) and a 6-degree-of-freedom couch. Optical surface imaging was used to monitor patient positioning during treatment.

Endpoint and analysis

**Figure 1:**
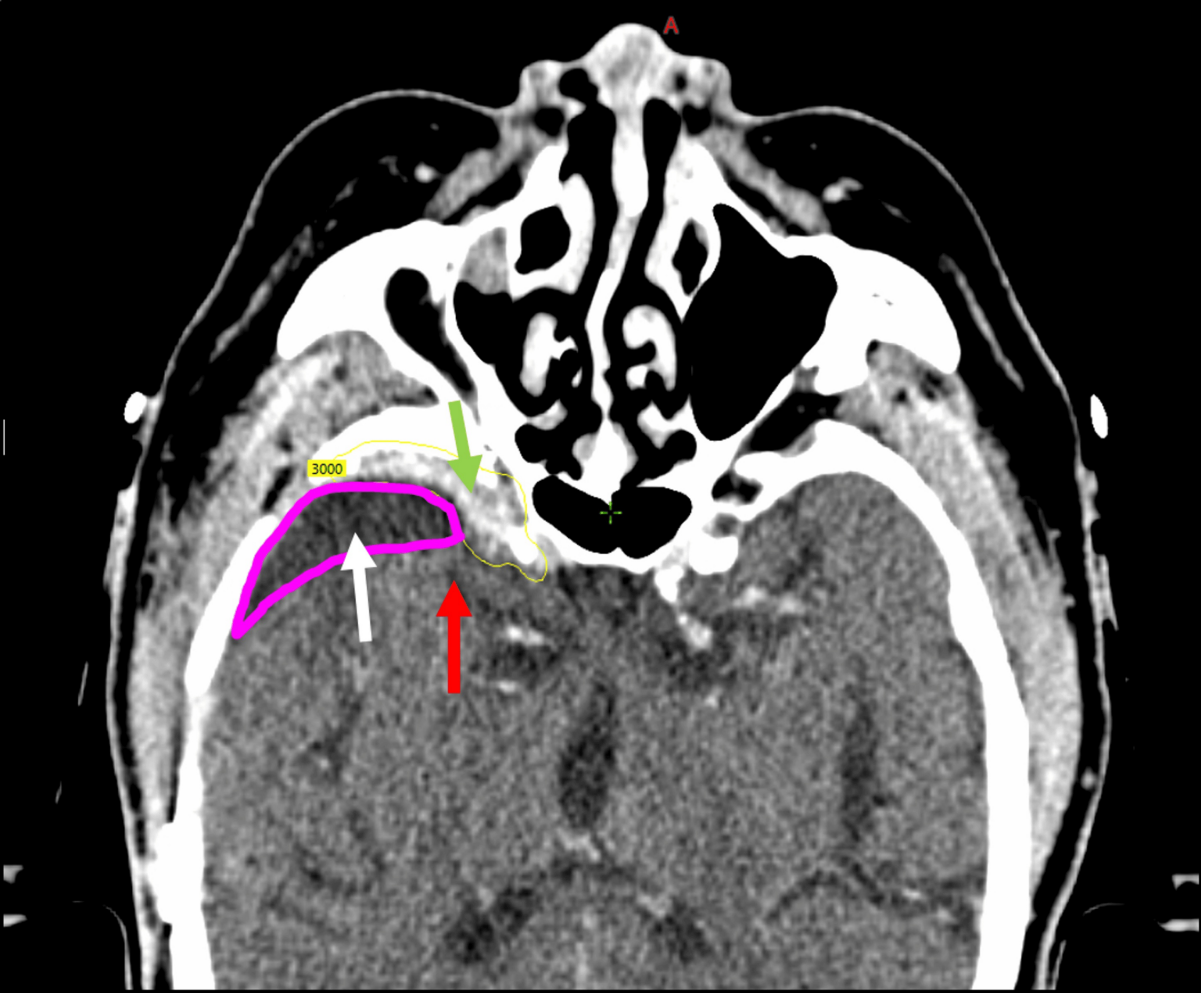
CT imaging showing tumor control endpoints. Recurrence within the radiation prescription isodose line was considered a local recurrence (green arrow), recurrence within the untreated cavity (magenta volume) was considered a cavity failure (white arrow), and recurrence within 2 cm of the cavity (regardless of treatment) and gross tumor volume was considered a marginal failure (red arrow).

Local recurrence was defined according to RECIST criteria as an increase of 20% in the greatest cross-sectional diameter on MRI (or CT, if MRI was contraindicated), with at least one voxel touching the prescribed treatment volume or any new tumor appearing after a GTR. To assess the role of the CTV margin, marginal recurrence was defined as any new lesion outside of the prescription volume but within 2 cm of the resection cavity. To evaluate the impact of elective cavity radiation, cavity recurrence was defined as any new lesion outside the prescription volume but within the resection cavity. Both cavity and marginal recurrences were determined on a per-patient basis. Figure 1 provides an example of potential failure classifications in a patient receiving 30 Gy in five fractions (yellow isodose line) to gross disease, without elective treatment of the resection cavity (magenta).

High-grade toxicity, as defined by CTCAE v5, was classified as an irreversible grade 3 or any grade 4 toxicity. Under this definition, conditions such as new headaches or seizures were not considered high-grade toxicity if they were medically managed without significant breakthroughs. Resection for suspected radionecrosis was categorized as both a local failure and high-grade toxicity if pathology confirmed the presence of a viable tumor.

Tumor control and high-grade toxicity rates were estimated using Kaplan-Meier survival analysis. Stratified variables were compared using a log-rank test. All analyses were conducted using IBM SPSS Statistics for Windows, Version 29 (Released 2023; IBM Corp., Armonk, NY, USA).

## Results

Between 2009 and 2022, 39 patients received 27.5-30 Gy in five fractions to their AM, with grading based on the WHO criteria active at the time of treatment. Patients and tumor characteristics are detailed in Table 2.

**Table 2:** Demographics of patients with grade 2 tumors evaluated.

Characteristic	N (%)
Gender	
Male	20 (51%)
Female	19 (49%)
Ki67%	
>10%	28 (60%)
<10%	6 (13%)
Unknown status	13 (27%)
Age (years)	
Average (range)	54 (14-80)
Deceased by time of study	
Yes	9 (24%)
No	29 (76%)
Resection status	
Gross total resection	16 (34%)
Subtotal resection	26 (55%)
Nondefinitive treatment	5 (11%)
ECOG performance status	
0	16 (41%)
1	15 (38%)
2	4 (10%)
3	3 (8%)
4	1 (3%)
High-grade toxicity	
Yes	2 (5%)
No	36 (95%)
Dose	
27.5 Gy	5 (11%)
30 Gy	42 (89%)
Meningioma volume (cc)	
Average (range)	11.95 (0.6-55.3)
Whole-cavity treatment	
Yes	36 (77%)
No	5 (10%)
No resection cavity	6 (13%)

The number of AMs treated post-STR, post-GTR recurrence, and definitively were 26, 16, and 5, respectively. The median follow-up for these patients was 32.5 months, with a range of 3.2-147.5 months. The anatomic location of each meningioma was recorded, following the typical distribution with an emphasis on higher-risk skull base locations.

**Figure 2:**
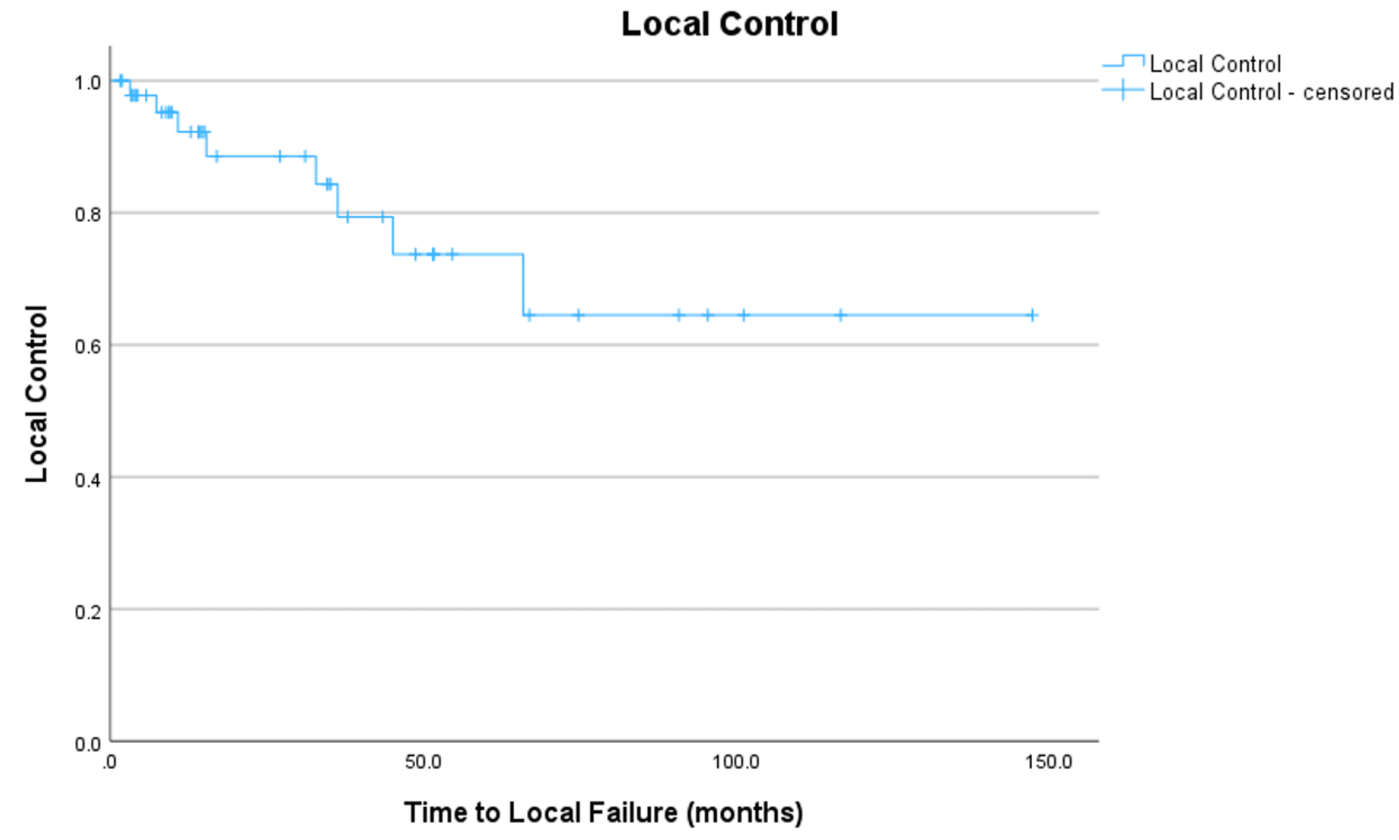
Kaplan-Meier estimates of local control in months for all atypical meningioma after FSRT with a three-year local control rate of 84%.

The estimated three-year local tumor control rate was 84% (Figure 2). 

**Figure 3:**
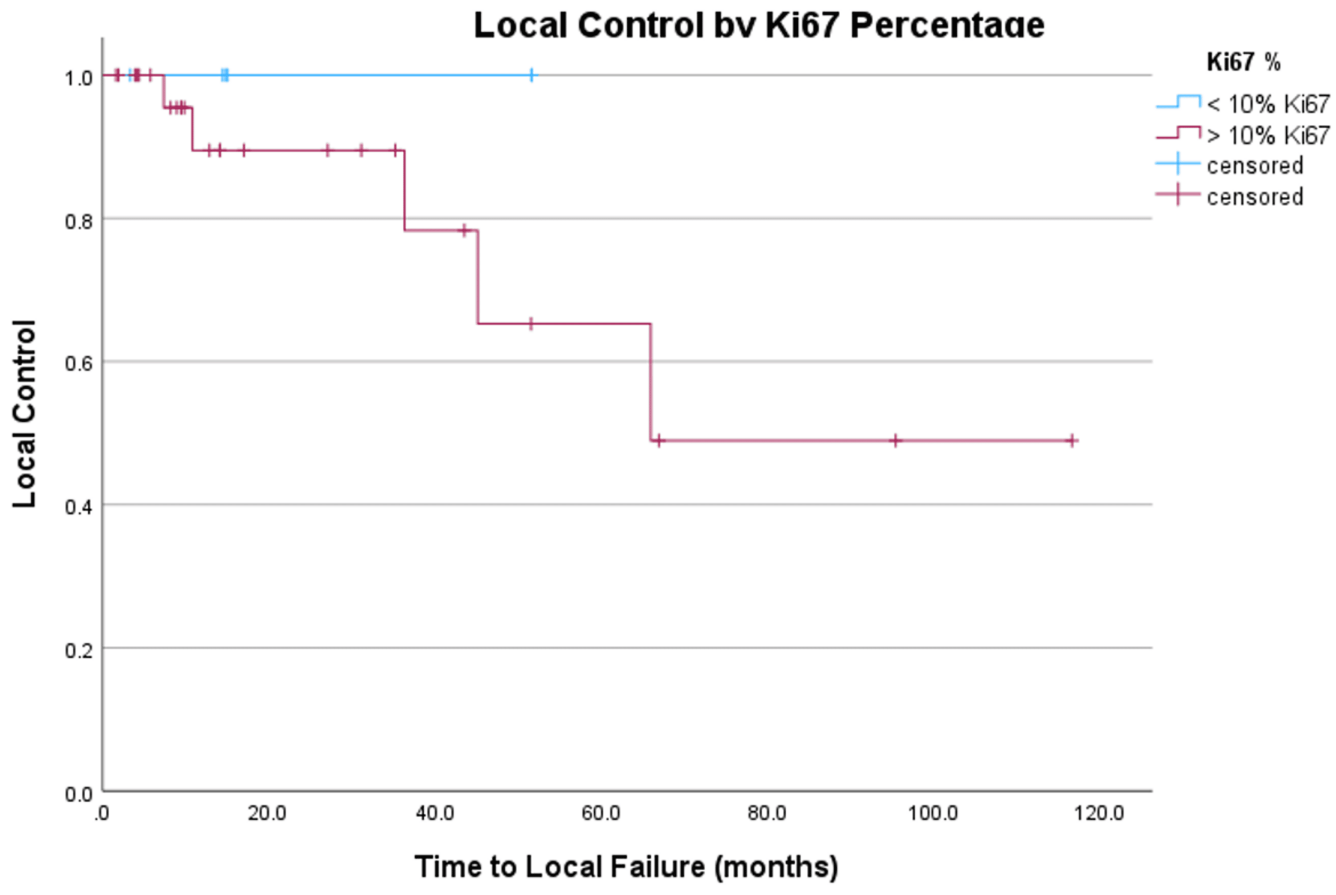
Kaplan-Meier comparison of local control in months of AM after FSRT stratified by Ki67 < 10% versus Ki67 > 10%. Lower Ki67 tumors are less likely to progress.

As expected, larger tumors were more likely to experience local failure (p < 0.001). Additionally, no local failures were observed in patients with a Ki67% < 10% (Figure 3).

**Figure 4:**
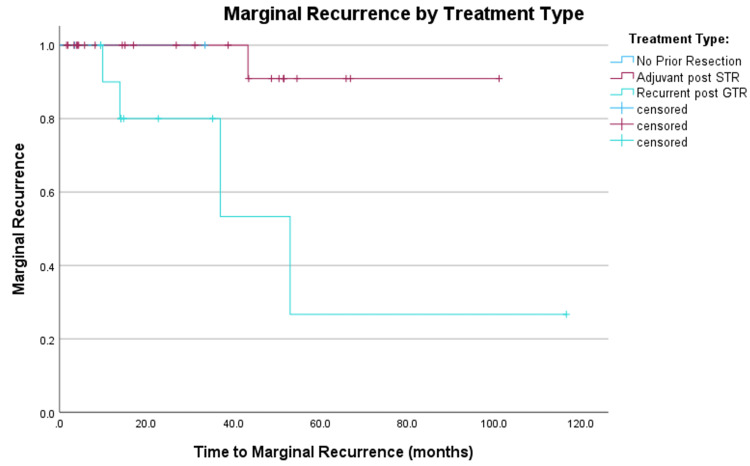
Kaplan-Meier comparison of failure marginal to treatment volume for patients treated with salvage post-GTR recurrence, with adjuvant post-STR, and definitively without prior resection. Recurrent tumors post-STR were more likely to fail marginally.

Two (5%) patients experienced high-grade toxicity, both of which were symptomatic radionecrosis requiring resection. Marginal control, defined as control outside the PTV but within 2 cm of the resection cavity, was estimated to be 92.3% at three years (Figure 4).

**Figure 5:**
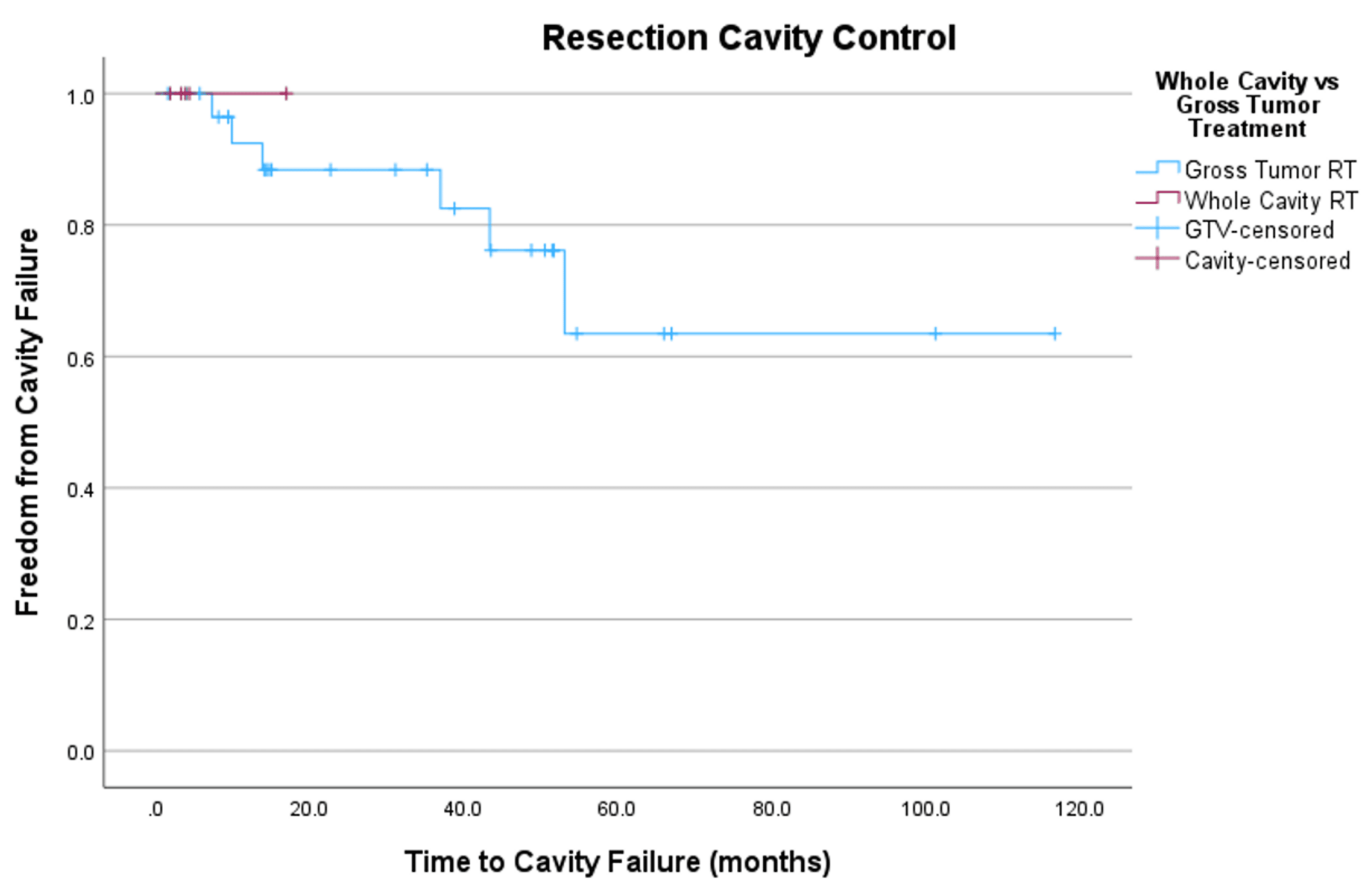
Kaplan-Meier comparison of in-cavity recurrence for patients treated to whole-cavity or gross residual tumor alone.

Cavity control, defined as the absence of new tumors within the resection cavity but outside the treated volume, was estimated to be 88% at three years for patients not receiving elective cavity radiation (Figure 5).

Of the five tumors treated to the entire resection cavity, none experienced cavity or marginal failure; however, follow-up for this subset of tumors was short, with a median of 3.9 months. Interestingly, recurrent tumors treated status post-GTR were more likely to experience marginal recurrence than tumors treated status post-STR (p = 0.009). Only one (4%) tumor treated status post-STR failed marginally, while four (33%) tumors treated status post-GTR recurrence failed.

## Discussion

The rate of high-grade toxicity in AMs receiving FSRT (27.5-30 Gy in five fractions) was low in this study. While improved on historical rates, local control suggests that dose escalation and longer-term follow-up may be necessary. Recurrent tumors appear more prone to marginal failures; however, further research is needed to identify which patients may benefit from additional CTV margins and which may benefit from more prolonged fractionated dosing schedules. Compared to the 1-2 cm CTV margins used in RTOG 0539, the CTVs in this study were far more selective, incorporating only the gross tumor with infrequent inclusion of cavity margins. In this study, the untreated resection cavity remained controlled, with 88% of patients free from new disease within the untreated cavity at three years. Despite the presence of a gross nodule in the cavity, this risk is lower than previously reported for observation of grossly resected AM cavities [18]. 

Stereotactic radiosurgery has previously been shown to provide modest improvements in local control. Prior studies have demonstrated five-year progression-free survival in AM of approximately 30%-40% with single-fraction radiosurgery [13,19]. EORTC 22042-26042 reported a three-year progression-free survival of 88.7% in patients with AM s/p GTR receiving 60 Gy of adjuvant radiation [9]. Our three-year progression-free survival of 84% compares favorably with these studies, considering our inclusion of higher-risk recurrent patients. Early experiences with gamma knife at UCSF for AM found a 73% freedom from local recurrence with salvage radiosurgery alone [6]. In terms of toxicity, single-fraction radiosurgery has previously been shown to have equivalent rates of symptomatic radionecrosis compared to conventional fractionation [20]. In the UCSF single-fraction salvage experience, 20% of patients experienced a CTCAE grade > 3 toxicity [6]. In this study, high-grade toxicity rates remain low when fractionated radiosurgery is used.

More recently, a multicenter retrospective analysis by Gallitto et al. highlighted the safety and efficacy of salvage SRS for recurrent grade 2 and 3 meningiomas, with a three-year progression-free survival of 57% in a cohort of 108 patients [21]. The 30% difference in local control between their study and our own is likely driven by the inclusion of grade 3 meningiomas in the study by Gallitto et al., given their reported hazard ratio of 6.80 for grade 3 histology. These tumors were excluded from our study. Both studies found 5% or less high-grade toxicity, supporting the safety of this treatment approach [21].

Several important questions involving the use of radiation with AM remain unanswered. These include the role of adjuvant radiation after GTR, optimal target volumes for radiation therapy, and how to incorporate PET imaging into adjuvant or salvage treatment decision-making.

Building on RTOG 0539, two studies are currently comparing adjuvant radiation with observation in AM status post-GTR. BN003 is a phase III prospective study comparing 59.4 Gy in 33 fractions vs observation in these patients [22]. The ROAM/EORTC-1308 study similarly compares 60 Gy in 30 fractions vs observation in gross totally resected patients with AM [23]. While informative, these studies neglect radiosurgery as an adjuvant treatment option. Future prospective studies should examine radiosurgery in addition to standard fractionation and address optimal CTV margin for adjuvant treatment for STR disease, especially with regard to elective cavity coverage.

The conformal and ablative nature of radiosurgery raises the question of optimal cavity margin in adjuvant and salvage radiation for these patients. In this study, marginal failure rates appear unacceptably high, especially in recurrent tumors s/p GTR. Options include the inclusion of the entire resection cavity in the CTV or targeting based on PET. ^68^Ga-DOTATE PET imaging better delineates meningioma volumes by targeting somatostatin receptors preferentially expressed by meningiomas [24]. This imaging could improve marginal failure rates in radiosurgery patients by better targeting residual disease, though more studies are needed. A small patient series from Ohio State demonstrated this by identifying previously occult marginal disease in almost 30% of patients with resected AM [25]. These results show the potential benefit of ^68^Ga-DOTATE PET in FSRT target delineation thereby reducing unnecessary treatment of normal marginal tissue.

As a single-institution retrospective review, our study is limited by a smaller cohort of patients and the potential for unidentified confounding variables. One such confounding variable in this study is the continued evolution of WHO classifications. In general, as more molecular variables have been considered in WHO classifications, many meningiomas that would historically have been considered grade 1 are now classified as grade 2. This highlights the importance of modern prospective studies with the inclusion of molecular classifiers for further stratification.

## Conclusions

The use of FSRT with 27.5-30 Gy in five fractions appears to be safe in the treatment of recurrent AM with acceptable toxicity. More studies are needed to determine whether the entire surgical cavity or only the gross volume of recurrence should be treated. FSRT also shows promise as an adjuvant therapy in AM treated after STR and should be compared to conventionally fractionated radiation and observation. The BN003 study aims to address the question of observation vs fractionated radiation after GTR, but more studies are needed to address FSRT in this setting. To date, no studies specifically examining FSRT for these patients are currently occurring. Future studies should focus on local control, toxicity, and whether treatment should target the gross tumor volume or the entire surgical cavity. Finally, incorporating ^68^Ga-DOTATATE PET into radiosurgery studies for AM may help reduce uncertainty in marginal treatment, potentially improving the detection of residual and recurrent disease and allowing for smaller treated volumes with less toxicity, without compromising cure rates.
